# Supplementary material for: Mapping the evolution of fertility support policies in China: A content and instrumental analysis
Source: PLoS One. 2025 Oct 9;20(10):e0332137. doi: 10.1371/journal.pone.0332137 (PMC12510515; doi:10.1371/journal.pone.0332137)
Supplement: S1 Appendix — (ZIP) [file pone.0332137.s001.zip › S1 Appendix. 226 original policy documents/91-劳动部关于发布《企业职工生育保险试行办法》的通知.doc]

劳动部关于发布《企业职工生育保险试行办法》的通知

**发布部门：** [劳动和社会保障部(含劳动部)(已撤销)](https://www.pkulaw.com/law/chl?Aggs.IssueDepartment=60212) [机构沿革](javascript:void(0)) 、

**发文字号：**劳部发〔1994〕504号

**发布日期：**1994.12.14

**实施日期：**1995.01.01

**时效性：** [现行有效](https://www.pkulaw.com/law/chl?Aggs.TimelinessDic=01)

**效力级别：** [部门规章](https://www.pkulaw.com/law/chl?Aggs.EffectivenessDic=XE0301)

**法规类别：** [工资福利与劳动保险](https://www.pkulaw.com/law/chl?Aggs.Category=08707)

　　　　　　劳动部关于发布《企业职工生育保险试行办法》的通知
　　　　　　　　　　 （劳部发〔１９９４〕５０４号）

　　为配合《[劳动法](https://www.pkulaw.com/chl/15fe2d94afee49a2bdfb.html)》的贯彻实施，更好地保障企业女职工的合法权益，我部制定了《企业职工生育保险试行办法》，现予发布，自１９９５年１月１日起试行。
　　　　　　　　　　　　　　　　　　　　　　　　　一九九四年十二月十四日
　　　　　　　　　　　　企业职工生育保险试行办法
　　第一条　为了维护企业女职工的合法权益，保障她们在生育期间得到必要的经济补偿和医疗保健，均衡企业间生育保险费用的负担，根据有关法律、法规的规定，制定本办法。

　　第二条　本办法适用于城镇企业及其职工。

　　第三条　生育保险按属地原则组织。生育保险费用实行社会统筹。

　　第四条　生育保险根据“以支定收，收支基本平衡”的原则筹集资金，由企业按照其工资总额的一定比例向社会保险经办机构缴纳生育保险费，建立生育保险基金。生育保险费的提取比例由当地人民政府根据计划内生育人数和生育津贴、生育医疗费等项费用确定，并可根据费用支出情况适时调整，但最高不得超过工资总额的百分之一。企业缴纳的生育保险费作为期间费用处理，列入企业管理费用。
　　职工个人不缴纳生育保险费。

　　第五条　女职工生育按照法律、法规的规定享受产假。产假期间的生育津贴按照本企业上年度职工月平均工资计发，由生育保险基金支付。

　　第六条　女职工生育的检查费、接生费、手术费、住院费和药费由生育保险基金支付。超出规定的医疗服务费和药费（含自费药品和营养药品的药费）由职工个人负担。
　　女职工生育出院后，因生育引起疾病的医疗费，由生育保险基金支付；其它疾病的医疗费，按照医疗保险待遇的规定办理。女职工产假期满后，因病需要休息治疗的，按照有关病假待遇和医疗保险待遇规定办理。

　　第七条　女职工生育或流产后，由本人或所在企业持当地计划生育部门签发的计划生育证明，婴儿出生、死亡或流产证明，到当地社会保险经办机构办理手续，领取生育津贴和报销生育医疗费。

　　第八条　生育保险基金由劳动部门所属的社会保险经办机构负责收缴、支付和管理。
　　生育保险基金应存入社会保险经办机构在银行开设的生育保险基金专户。银行应按照城乡居民个人储蓄同期存款利率计息，所得利息转入生育保险基金。

　　第九条　社会保险经办机构可从生育保险基金中提取管理费，用于本机构经办生育保险工作所需的人员经费、办公费及其它业务经费。管理费标准，各地根据社会保险经办机构人员设置情况，由劳动部门提出，经财政部门核定后，报当地人民政府批准。管理费提取比例最高不得超过生育保险基金的百分之二。
　　生育保险基金及管理费不征税、费。

　　第十条　生育保险基金的筹集和使用，实行财务预、决算制度，由社会保险经办机构作出年度报告，并接受同级财政、审计监督。

　　第十一条　市（县）社会保险监督机构定期监督生育保险基金管理工作。

　　第十二条　企业必须按期缴纳生育保险费。对逾期不缴纳的，按日加收千分之二的滞纳金。滞纳金转入生育保险基金。滞纳金计入营业外支出，纳税时进行调整。

　　第十三条　企业虚报、冒领生育津贴或生育医疗费的，社会保险经办机构应追回全部虚报、冒领金额，并由劳动行政部门给予处罚。
　　企业欠付或拒付职工生育津贴、生育医疗费的，由劳动行政部门责令企业限期支付；对职工造成损害的，企业应承担赔偿责任。

　　第十四条　劳动行政部门或社会保险经办机构的工作人员滥用职权、玩忽职守、徇私舞弊，贪污、挪用生育保险基金，构成犯罪的，依法追究刑事责任；不构成犯罪的，给予行政处分。

　　第十五条　省、自治区、直辖市人民政府劳动行政部门可以按照本办法的规定，结合本地区实际情况制定实施办法。

　　第十六条　本办法自１９９５年１月１日起试行。
